# Effect of Tendon-Related Variables on the Behavior of Externally CFRP Prestressed Concrete Beams

**DOI:** 10.3390/ma16145197

**Published:** 2023-07-24

**Authors:** Tiejiong Lou, Han Hu, Miao Pang

**Affiliations:** 1 School of Civil Engineering and Architecture, Wuhan University of Technology, Wuhan 430070, China; loutiejiong@dec.uc.pt (T.L.);; 2 CEMMPRE, ARISE, Department of Civil Engineering, University of Coimbra, 3030-788 Coimbra, Portugal; 3 Department of Civil Engineering, Zhejiang University, Hangzhou 310058, China

**Keywords:** external prestressing, CFRP tendon, flexural strength, tendon stress, numerical modeling, analytical model

## Abstract

This work assesses the flexural performance of prestressed concrete beams with external carbon fiber-reinforced polymer (CFRP) tendons, focusing on tendon-related variables. A finite element analysis (FEA) method is verified. A numerical parametric analysis of prestressed concrete beams with external CFRP tendons is carried out. Four tendon-related variables are considered, namely, the area, initial prestress, depth and elastic modulus of tendons. The analysis shows that flexural ductility decreases as the tendon area, initial prestress or elastic modulus increases but is insensitive to the tendon depth. The ultimate tendon stress increment (Δ*σ_p_*) is influenced by all of the four variables investigated. JGJ 92-2016 (Chinese technical specification for concrete structures prestressed with unbonded tendons) significantly underestimates Δ*σ_p_* and, hence, is over-conservative for the strength design of these beams. An equation is proposed for calculating Δ*σ_p_*, taking into account all four variables investigated. An analytical model is then developed to estimate the flexural strength (*M_u_*) of prestressed concrete beams with external CFRP tendons. The proposed analytical model shows good agreement with FEA, i.e., the mean discrepancy for Δ*σ_p_* is 0.9% with a standard deviation of 11.1%; and the mean discrepancy for *M_u_* is −1.6% with a standard deviation of 2.1%.

## 1. Introduction

External prestressing is a powerful technique for structural rehabilitation and construction [1,2]. The major concern for this technique is the corrosive damage of external tendons made of conventional prestressing steel. The utilization of fiber-reinforced polymer (FRP) reinforcement in engineering is widespread [3,4]. The composite reinforcement may be made of glass fiber-reinforced polymer (GFRP) [5], aramid fiber-reinforced polymer (AFRP) [6], basalt fiber-reinforced polymer (BFRP) [7] or carbon fiber-reinforced polymer (CFRP) [8]. Of various FRP composites, carbon fiber-reinforced polymer (CFRP) possesses the best resistance to creep, namely, it can sustain around 80% of the tensile strength without experiencing creep rupture [9,10]. Therefore, CFRP is recognized to be ideal for prestressing applications [11,12,13]. This non-corrosive composite material is particularly suitable for external tendons in lieu of conventional prestressing steel in overcoming the corrosive problem [14,15], as these tendons are exposed to harsh conditions.

Researchers have paid much attention to prestressed concrete beams with external CFRP tendons. Grace et al. [16] performed a set of laboratory tests including one reference box-specimen without external tendons, one box-specimen prestressed by external CFRP tendons and one box-specimen with external CFRP tendons without prestressing. Their tests showed that the prestressing of girders by external CFRP tendons significantly increased the ultimate load-carrying capacities but reduced the ultimate deflection and flexural ductility. Bennitz et al. [17] tested seven reinforced concrete T-beams including one reference specimen without external prestressing and six specimens prestressed with external CFRP tendons. The main investigated variables were the deviator configuration (no deviators or one deviator at midspan), initial tendon depth and initial prestress level. Some of the specimens were designed based on the specimens that were prestressed with external steel tendons and tested by Tan and Ng [18] (i.e., using CFRP tendons instead of steel tendons). Similar to the findings concluded by Grace et al. [16], the test results demonstrated that beams with external CFRP tendons experienced higher flexural strength and lower ductility than the reference beam. In addition, beams with external CFRP tendons exhibited similar behavior compared to the counterparts with external steel tendons. The slight performance difference between beams with external CFRP and steel tendons was attributed to their difference in the tendon modulus of elasticity. This observation was later confirmed by a numerical work in terms of comprehensive aspects of behavior, including deformation, neutral axis evolution, tendon stress and flexural strength [19]. Contrary to the limited influence of the external tendon type, a numerical study performed by Pang et al. [20] showed that using FRP rebars instead of steel rebars significantly affected the structural behavior of concrete beams prestressed with external CFRP tendons.

Although much investigation has been performed, knowledge about externally CFRP prestressed beams has yet to be further developed. For example, the prestressing tendons are the primary material controlling the overall behavior of externally post-tensioned beams. Variables related to CFRP tendons include the tendon area, prestress level, tendon depth and modulus of elasticity. Note that the CFRP modulus of elasticity covers a large range and may vary from 80 to 500 GPa according to [10]. So far, the effects of these variables on the flexural response have not been fully addressed.

In this paper, a finite element analysis (FEA) method is verified with experimental data. A numerical study is carried out to examine the effects of various tendon-related variables. Based on the results of numerical parametric analysis, an analytical model is developed to predict the ultimate tendon stress and the flexural strength of prestressed concrete beams with external CFRP tendons.

## 2. Materials, Method and Verification

### 2.1. Materials and Method

According to [21], the stress–strain (*σ_c_*–*ε_c_*) law for compressive concrete is represented by

(1) $$\frac{\sigma_{c}}{f_{ck}+8}=\frac{k(\varepsilon_{c}/\varepsilon_{0})-{\left(\varepsilon_{c}/\varepsilon_{0}\right)}^{2}}{1+(k-2)(\varepsilon_{c}/\varepsilon_{0})}$$

where *f_ck_* is the cylinder compressive strength; *k* is a coefficient and *ε_c_*_0_ is the strain corresponding to peak stress.

According to [22], the stress–strain law for tensile concrete is represented by

(2) $$\sigma_{c}=\left\{\begin{array}{c} E_{c}\varepsilon_{c}\;\mathrm{for}\;\varepsilon_{c}\leq \varepsilon_{cr}\\ f_{t}\left(\frac{\varepsilon_{u}-\varepsilon_{c}}{\varepsilon_{t0}-\varepsilon_{cr}}\right)\;\mathrm{for}\;\varepsilon_{cr}<\varepsilon_{c}\leq \varepsilon_{t0}\\ 0\;\mathrm{for}\;\varepsilon_{c}>\varepsilon_{t0} \end{array}\right.$$

where *E_c_* is the concrete elastic modulus; *f_t_* is the tensile strength; *ε_c_*_r_ is the cracking strain and *ε_t_*_0_ = 10*ε_c__r_*.

The stress–strain (*σ_p_*–*ε_p_*) law for CFRP prestressing tendons [10] is represented by

(3) $$\sigma_{p}=\left\{\begin{array}{c} E_{p}\varepsilon_{p}\;\mathrm{for}\;\varepsilon_{p}\leq \varepsilon_{pu}\\ 0\;\mathrm{for}\;\varepsilon_{p}>\varepsilon_{pu} \end{array}\right.$$

where *E_p_* is the tendon elastic modulus and *ε_pu_* is the ultimate tendon strain.

It is assumed that the stress–strain (*σ_s_*–*ε_s_*) law for steel bars [10] is represented by

(4) $$\sigma_{s}=\left\{\begin{array}{c} E_{s}\varepsilon_{s}\;\mathrm{for}\;\varepsilon_{s}\leq \varepsilon_{y}\\ 0\;\mathrm{for}\;\varepsilon_{s}>\varepsilon_{y} \end{array}\right.$$

where *E_s_* is the steel elastic modulus and *ε_y_* is the yield strain.

An FEA method for prestressed beams with external tendons was developed [23]. The following assumptions are adopted: (1) plane section remaining plane (excluding external tendons due to their unbonded nature [24]); (2) negligible bond-slip for internal steel bars; (3) negligible shear deformation (given that prestressed concrete beams generally have a high slenderness); (4) negligible frictional loss between external tendons and deviators (this assumption is reasonable in practice but may lead to a bit of an overestimation of the tendon stress). The Euler–Bernoulli beam theory is employed. The material laws for concrete and steel bars are introduced by applying the layered method. External prestressing is transformed into equivalent loads. The analysis consists of two steps. The first step is to determine the stress and deformation at self-weight only. The second step is to perform the full-range analysis from zero loads up to failure. During the analysis, as long as concrete crushing or reinforcement rupture occurs, the beam fails. Details of the FEA method were reported in [23].

### 2.2. Verification

**Figure 1:**
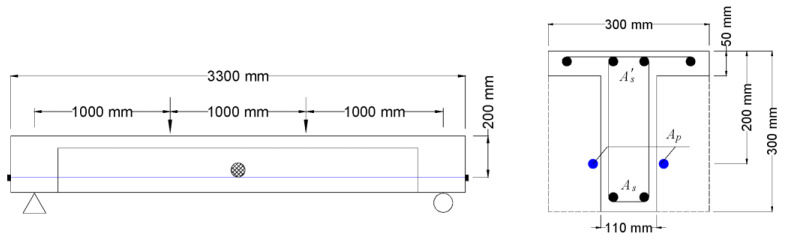
Specimens prestressed with external CFRP tendons [17].

Two beam specimens (B4 and B5) are selected herein [17]. The T-beams had a clear span of 3000 mm, subjected to two concentrated loads at third points, as illustrated in Figure 1. External tendons were attached with one deviator at the midspan. The specimens were designed to be identical except for the prestress level, i.e., the initial prestress was 396 MPa for B4 and 889 MPa for B5. CFRP tendons had an area of 100.5 mm^2^, an elastic modulus of 158 GPa and a rupture strength of 2790 MPa. The bottom steel bars had an area of 402 mm^2^, an elastic modulus of 172 GPa and a yield strength of 560 MPa. The top steel bars had an area of 201 mm^2^, an elastic modulus of 187 GPa and a yield strength of 510 MPa. The targeted *f_ck_* was 30 MPa.

**Figure 2:**
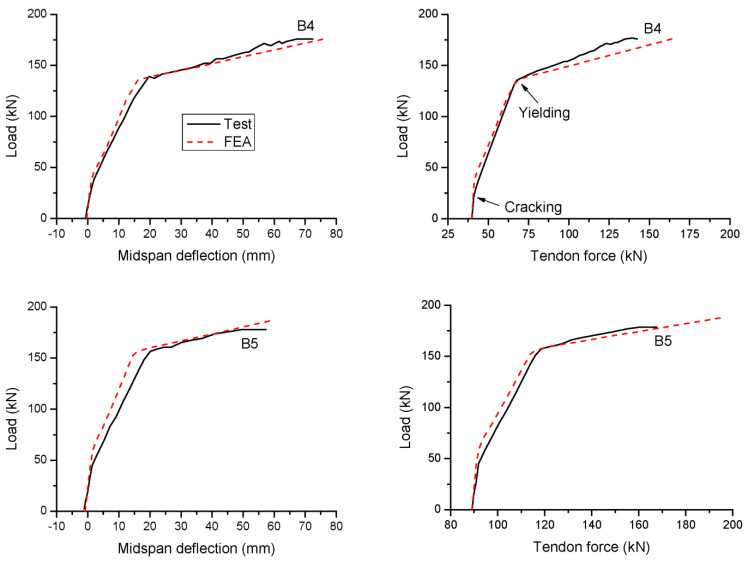
Comparison with FEA results for test specimens.

A comparison between the test and FEA results regarding the load versus deflection and load versus tendon force for two specimens is illustrated in Figure 2. It is seen that the FEA is able to reasonably capture the key response characteristics of the specimens during the complete loading stages, including the cracking, yielding and ultimate limit states. In addition, both test and FEA results demonstrate that Specimen B5 exhibits higher cracking and ultimate loads but a lower ultimate deflection and an increase in the tendon force compared to Specimen B4. This observation indicates the importance of the prestress level. In the following section, this variable is analyzed in more detail along with other important variables related to CFRP tendons (tendon area, depth and elastic modulus)

## 3. Numerical Study

**Figure 3:**
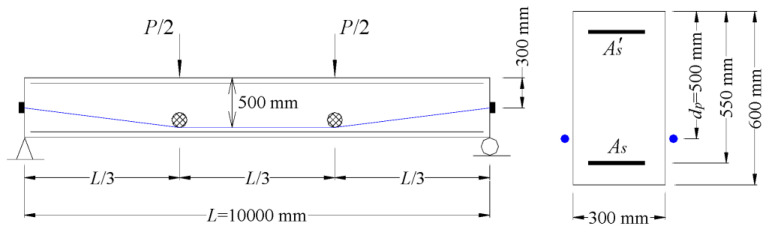
Externally prestressed concrete reference beam for investigation.

Figure 3 shows an externally prestressed concrete reference beam for numerical parametric study. The *f_ck_* is 60 MPa. The tendon area is 1100 mm^2^. CFRP tendons have a rupture strength of 1840 MPa, an elastic modulus of 150 GPa and an initial prestress of 1104 MPa. Either the bottom or top steel bars have a cross-sectional area of 360 mm^2^, yield strength of 450 MPa and elastic modulus of 200 GPa.

### 3.1. Effect of Tendon Area

**Figure 4:**
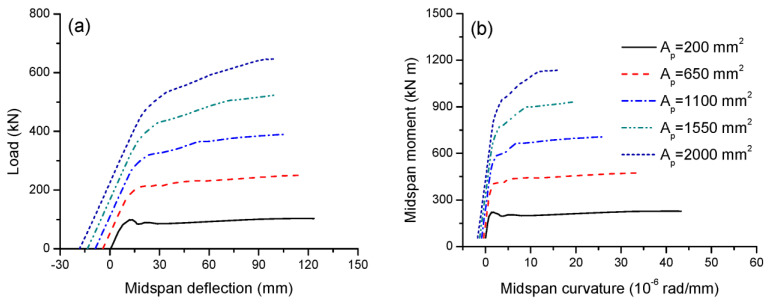
Effect of tendon area on the deformation development. (**a**) Load-deflection; (**b**) moment-curvature.

The tendon area, *A_p_*, varies between 200 and 2000 mm^2^ to examine its effect on the structural response. Figure 4a shows that, at a small area of 200 mm^2^, the prestressing effect is not prominent, just counteracting the effect of the self-weight, i.e., the initial deflection prior to the live load is around zero. At *A_p_* = 650 mm^2^ or above, the prestressing effect becomes pronounced, leading to an upward deflection at the initial state. However, at high areas of 1550 and 2000 mm^2^, there occur excessive prestressing effects. In these cases, the top face of the critical section has cracked prior to load application, and the upward deflection is rather big (13.5 and 18.3 mm). It is observed in Figure 4a that as the tendon area increases, the cracking and ultimate loads significantly increase, while the ultimate deflection obviously decreases. In this analysis, increasing the tendon area from 200 to 2000 mm^2^ leads to an increase in the ultimate load by 528.5% and a decrease in the ultimate deflection by 18.3%. Figure 4b shows that, at the initial state, the self-weight moment is 56 kN·m. A higher tendon area causes substantially higher flexural stiffness and flexural strength but a markedly smaller ultimate curvature.

The flexural ductility can be expressed by either deflection or curvature ductility, defined as the ratio of deflection or curvature at ultimate to that at yielding. Table 1 presents the data for the flexural ductility of beams with different tendon areas. It is seen that the flexural ductility quickly deceases as the tendon area increases, i.e., increasing the tendon area from 200 to 2000 mm^2^ leads to a decrease in deflection ductility by 70.8% and curvature ductility by 73.9%.

**Figure 5:**
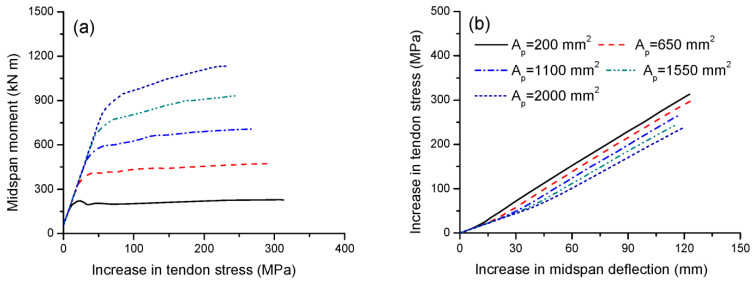
Effect of tendon area on tendon stress development. (**a**) Moment versus increase in tendon stress; (**b**) increase in deflection versus increase in tendon stress.

Figure 5a,b illustrate the tendon stress versus midspan moment and deflection for beams with various tendon areas, respectively. In the initial range of loading up to tensile cracking, the rate of increase in tendon stress with the moment is independent of the tendon area, as demonstrated in Figure 5a. A higher tendon area causes a higher cracking moment and, correspondingly, a larger increase in tendon stress at cracking. Moreover, the ultimate stress increase in tendons decreases as the tendon area increases. In this analysis, increasing the tendon area from 200 to 2000 mm^2^ reduces the ultimate stress increase in external tendons by 24.4%. Figure 5b demonstrates that, for the same deflection, a high tendon area causes a small increase in tendon stress. The slopes of the stress–deflection relationship for beams with *A_p_* = 200, 650, 1100, 1550 and 2000 mm^2^ are 2.54, 2.50, 2.45, 2.39 and 2.35 MPa/mm, respectively.

### 3.2. Effect of Prestress Level

**Figure 6:**
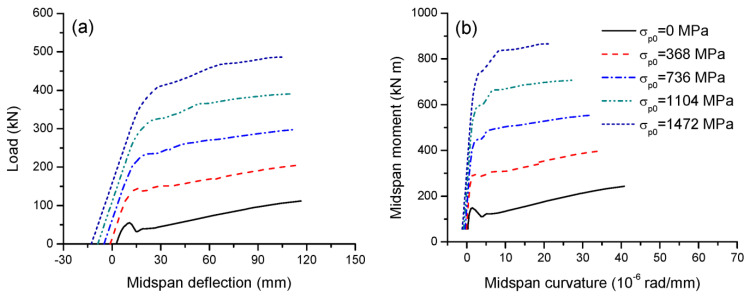
Effect of initial prestress on the deformation development. (**a**) Load-deflection; (**b**) moment-curvature.

Five initial prestress levels are considered, i.e., 0, 20, 40, 60 and 80% of the rupture strength, or, equivalently, the initial prestress, *σ_p_*_0_, corresponds to 0, 368, 736, 1104 and 1472 MPa. Figure 6a,b show the load-deflection and moment-curvature behavior of beams with various prestress levels.

The initial deflection is highly dependent on the prestress level, i.e., a higher prestress level corresponds to a larger upward deflection. At a prestress level of zero, there is a downward deflection due to the self-weight effect, as expected. In addition, a higher prestress level effectively improves not only the cracking load and moment but also the ultimate load and flexural strength. It is worth mentioning that this observation is different from the case using bonded tendons. Generally, bonded FRP tendons rupture at failure, and therefore, the prestress level has practically no influence on the ultimate load or flexural strength of the beams [25]. On the other hand, the stress increase in external tendons is much slower than that in bonded tendons due to strain incompatibility between external tendons and the adjacent concrete and also the second-order effects [26]. Therefore, the ultimate stress in external tendons is generally below their rupture strength. The ultimate load or flexural strength is controlled by the tendon stress and is therefore strongly dependent on the prestress level in external tendons. Moreover, a higher prestress level results in a substantially higher flexural stiffness and lower ultimate curvature, while this variable appears to have a marginal effect on the ultimate deflection. Table 2 presents the data for flexural ductility for beams with different prestress levels. It is seen that as the prestress level increases, the flexural ductility quickly decreases. Increasing the initial prestress from 0 to 1472 MPa leads to a decrease in deflection ductility by 70.3% and in curvature ductility by 60.9%.

**Figure 7:**
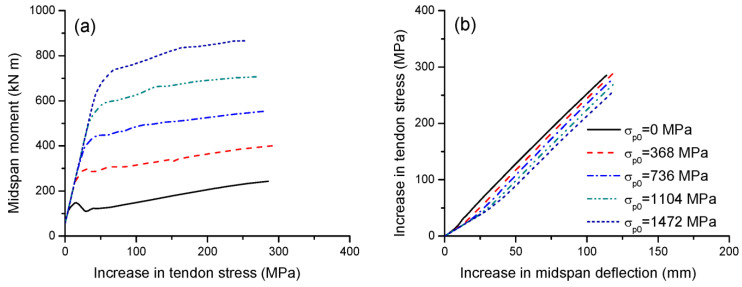
Effect of initial prestress on tendon stress development. (**a**) Moment versus increase in tendon stress; (**b**) increase in deflection versus increase in tendon stress.

Figure 7a,b illustrate the effect of the initial prestress on the tendon stress versus midspan moment and deflection, respectively. Over the elastic range, the initial prestress has no impact on the increase in tendon stress as the moment develops, as shown in Figure 7a. A higher initial prestress corresponds to a higher increment in tendon stress at cracking but, generally, a lower one at ultimate. In this analysis, increasing the prestress level from 20 to 80% causes a reduction in the ultimate stress increase in external tendons by 10.7%. It is seen in Figure 7b that, when deflecting, the greater the initial prestress, the slower the increase in tendon stress.

### 3.3. Effect of Tendon Depth

**Figure 8:**
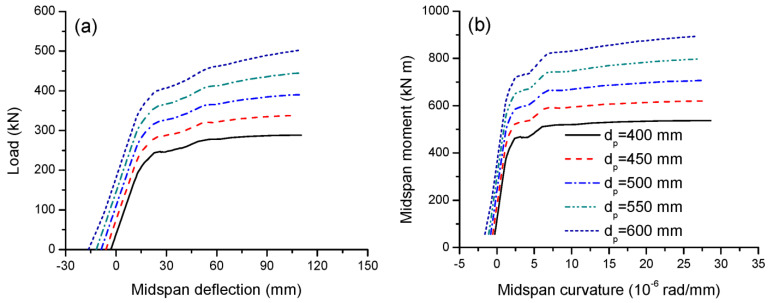
Effect of tendon depth on the deformation development. (**a**) Load-deflection; (**b**) moment-curvature.

The initial tendon depth (maximum depth), *d_p_*, ranging from 400 to 600 mm, is used to investigate its effect on the structural response. The load-deflection and moment-curvature behavior are shown in Figure 8a,b, respectively. From Figure 8, it is seen that a larger tendon depth registers a larger upward deflection prior to load application. In addition, the cracking and ultimate loads or moments substantially increase as the tendon depth increases. In this analysis, increasing the tendon depth from 400 to 600 mm^2^ causes an increase in the ultimate load by 74.2%. However, the ultimate deflection, curvatures and flexural ductility are insensitive to the tendon depth, as presented in Table 3.

**Figure 9:**
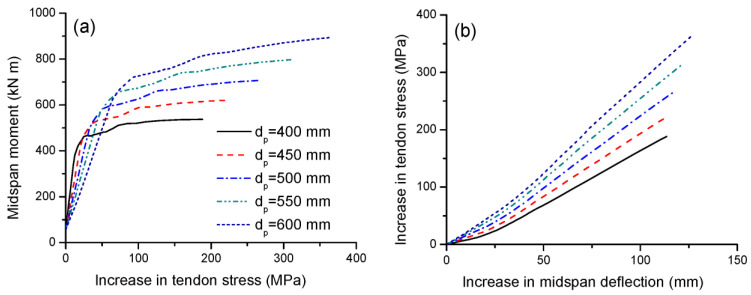
Effect of tendon depth on tendon stress development. (**a**) Moment versus increase in tendon stress; (**b**) increase in deflection versus increase in tendon stress.

Figure 9a,b illustrate the tendon stress versus midspan moment and deflection for beams with various tendon depths, respectively. As the tendon depth increases, the ultimate tendon stress increases substantially. As mentioned previously, the beams with different tendon depths exhibit approximately the same deformation at ultimate, implying there are approximately the same concrete strain distributions along the depth of the sections of these beams. Therefore, the larger the tendon depth, the larger the average change in the concrete strain at the same level of the external tendons and, thereby, the larger the tendon stress increase. Due to a lower reduction in the effective tendon depth, a larger tendon depth assumes relatively less significant second-order effects, which would reduce relatively less tendon stresses. In this analysis, increasing the tendon depth from 400 to 600 mm causes an increase in the ultimate tendon stress increment by 93.2%. For the same deflection, the greater the tendon depth, the greater the tendon stress increase. The slopes of the stress–deflection relationship for beams with *d_p_* = 400, 450, 500, 550 and 600 mm are 1.70, 1.96, 2.45, 2.86 and 3.30 MPa/mm, respectively.

### 3.4. Effect of Tendon Elastic Modulus

**Figure 10:**
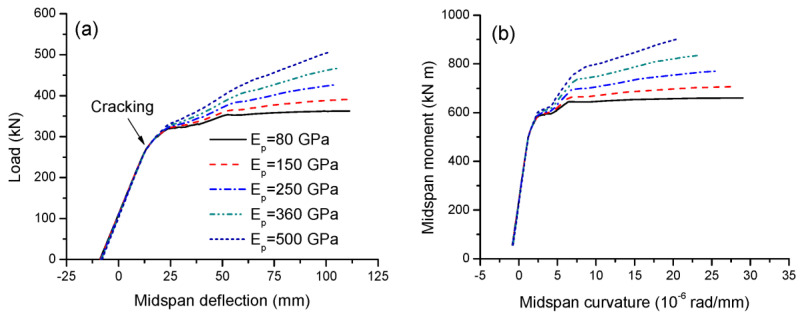
Effect of tendon elastic modulus on the deformation development. (**a**) Load-deflection; (**b**) moment-curvature.

The elastic modulus of CFRP tendons, *E_p_*, ranges from 80 to 500 GPa [10]. Figure 10a,b show the effect of the tendon elastic modulus on the load-deflection and moment-curvature behavior, respectively. The beams exhibit identical behavior up to cracking. After that, the behaviors differ because the contribution of the external tendon becomes increasingly important. A higher tendon modulus of elasticity mobilizes stiffer behavior of the beams. As the tendon modulus of elasticity increases, the ultimate load and flexural strength increase, while the ultimate deflection and curvature tend to decrease. As presented in Table 4, increasing the tendon elastic modulus from 80 to 500 GPa leads to a decrease in deflection ductility by 11.1% and in curvature ductility by 31.4%.

**Figure 11:**
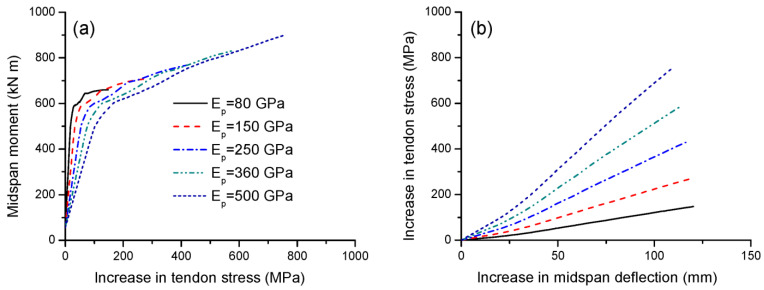
Effect of tendon elastic modulus on tendon stress development. (**a**) Moment versus increase in tendon stress; (**b**) increase in deflection versus increase in tendon stress.

Figure 11a,b show the effect of the tendon elastic modulus on the tendon stress versus midspan moment and deflection, respectively. At a given moment or deflection level, a high tendon elastic modulus corresponds to a significantly higher tendon stress. The ultimate stress increase in external tendons with an elastic modulus of 500 GPa is 5.17 times that of 80 GPa. It should be noted that the stress ratio of 5.17 is smaller than the modulus ratio of 6.25, as a higher tendon elastic modulus would lead to a smaller ultimate tendon strain. The slopes of the stress–deflection relationship for beams with *E_p_* = 80, 150, 250, 360 and 500 GPa are 1.33, 2.45, 3.98, 5.57 and 7.43 MPa/mm, respectively.

## 4. Analytical Study

### 4.1. Method

In externally post-tensioned members, the ultimate tendon stress, *σ_pu_*, depends on the whole member deformation, and therefore, its accurate predictions are complicated. The calculation of *σ_pu_* is essential for the flexural strength design of externally prestressed members [27,28,29,30,31,32]. The stress *σ_pu_* is commonly expressed by

(5) $$\sigma_{pu}=\sigma_{pe}+\Delta \sigma_{p}$$

where Δ*σ_p_* is the ultimate stress increment and *σ_pe_* is the effective prestress.

JGJ 92-2016 [33] recommended the following equation for predicting Δ*σ_p_*:

(6) $$\Delta \sigma_{p}=(240-335\omega_{0})(0.45+5.5h/L)k_{1}$$

where *ω*_0_ is the combined reinforcing index; *h* is the section height; *L* is the span and *k*_1_ is a coefficient associated with the pattern of loading on continuous beams. For the simply supported beams considered in this study, *k*_1_ = 1.0. The *ω*_0_ is expressed by

(7) $$\omega_{0}=\frac{A_{p}\sigma_{pe}+A_{s}f_{y}}{bd_{p}f_{ck}}$$

where *b* is the section width and *A_s_* and *f_y_* are the tensile steel bar area and yield strength, respectively.

**Figure 12:**
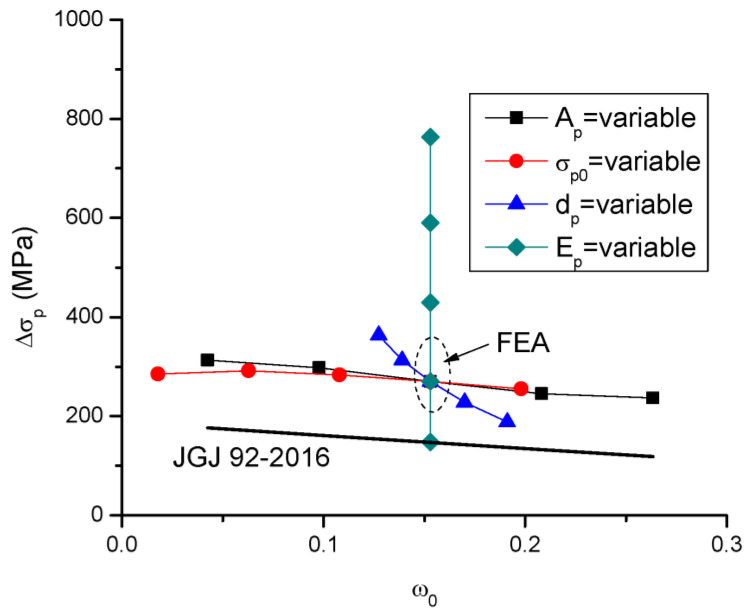
Relationships between Δ*σ_p_* and *ω*_0_ according to FEA and JGJ 92-2016.

Figure 12 shows the Δ*σ_p_*–*ω*_0_ relationships for the beams with different tendon-related variables obtained by FEA along with the JGJ 92-2016 curve. By adopting the key parameter *ω*_0_, JGJ 92-2016 takes into account the effect of the tendon area, prestress level and tendon depth but neglects the effect of the tendon modulus of elasticity. In addition, JGJ 92-2016 significantly underestimates the value of Δ*σ_p_*, except for the beam with *E_p_* = 80 GPa.

**Figure 13:**
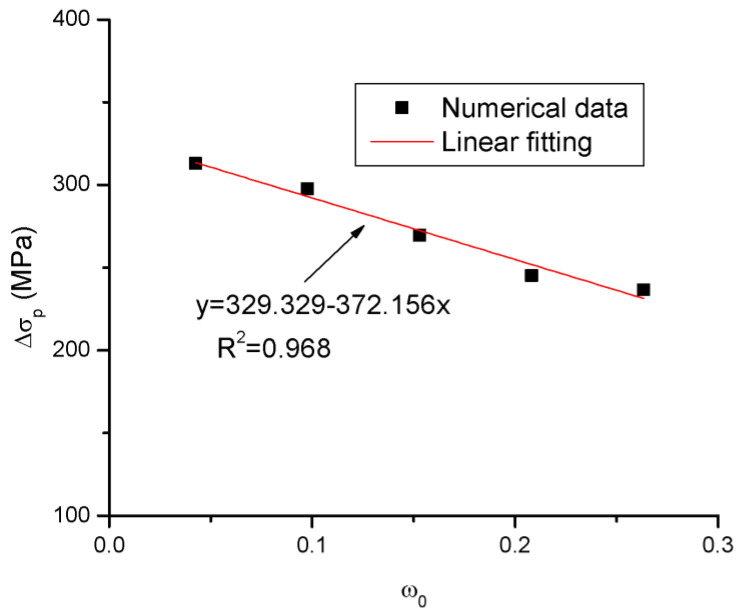
Fitting to FEA data about the Δ*σ_p_*–*ω*_0_ relationship.

The numerical analysis demonstrates that the ultimate tendon stress is affected by all four tendon-related variables, i.e., the tendon area, prestress level, tendon depth and modulus of elasticity. As the parameter *ω*_0_ involves three of the tendon-related variables (i.e., *A_p_*, *d_p_* and *σ_pe_*), this parameter is adopted in a new equation to be developed herein for the prediction of Δ*σ_p_*. As illustrated in Figure 13, fitting to the FEA data about the Δ*σ_p_*–*ω*_0_ relationship of beams with various tendon areas leads to the following expression:

(8) $$\Delta \sigma_{p}=330-372\omega_{0}$$

**Figure 14:**
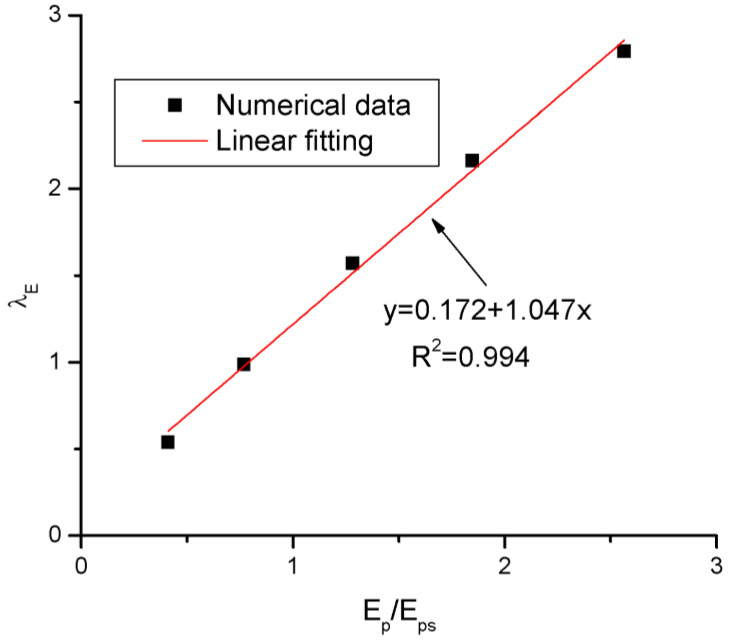
Fitting to FEA data about the *λ_E_* versus *E_P_*/*E_ps_* relationship.

Note that the above equation does not consider the tendon modulus of elasticity, which has been demonstrated to be crucial for Δ*σ_p_*. In order to include the effect of the tendon elastic modulus, Equation (8) is modified by introducing a coefficient as follows:

(9) $$\Delta \sigma_{p}=\lambda_{E}(330-372\omega_{0})$$

where *λ_E_* is a coefficient related with the tendon elastic modulus *E_p_*. The FEA data regarding the variation in *λ_E_* against *E_p_*/*E_ps_* is presented in Figure 14, where *E_ps_* is the elastic modulus of prestressing steel, taken to be equal to 195 GPa. According to the fit curve, *λ_E_* is expressed by

(10) $$\lambda_{E}=0.172+1.047\left(\frac{E_{p}}{E_{ps}}\right)$$

The axial equilibrium of beams is given by the following equation [34]:

(11) $$0.85f_{ck}b\beta_{1}c_{u}=A_{p}\sigma_{pu}+A_{s}f_{y}-A_{s}^{\prime }f_{y}^{\prime }$$

where *β*_1_ = 0.85; $A_{s}^{\prime }$ and $f_{y}^{\prime }$ are the compressive steel bar area and yield strength, respectively and *c_u_* is the neutral axis depth. According to Equation (11), *c_u_* is calculated by

(12) $$c_{u}=\frac{A_{p}\sigma_{pu}+A_{s}f_{y}-A_{s}^{\prime }f_{y}^{\prime }}{0.85f_{ck}b\beta_{1}}$$

The flexural strength is determined by the following equation [34]:

(13) $$M_{u}=A_{p}\sigma_{pu}d_{e}+A_{s}f_{y}d_{s}-A_{s}^{\prime }f_{y}^{\prime }d_{s}^{\prime }-0.85f_{ck}b{\left(\beta_{1}c_{u}\right)}^{2}/2$$

where *d_s_* and $d_{s}^{\prime }$ are the depths of tensile and compressive bars, respectively, and *d_e_* is the effective depth of external tendons, which is given by

(14) $$d_{e}=R_{d}d_{p}$$

where *R_d_* is a reduction coefficient due to second-order effects. According to [35], for third-point loading, the value of *R_d_* is calculated from

(15) $$R_{d}=1.25-0.01(L/d_{p})-0.38(S_{d}/L)\leq 1.0$$

### 4.2. Results

**Figure 15:**
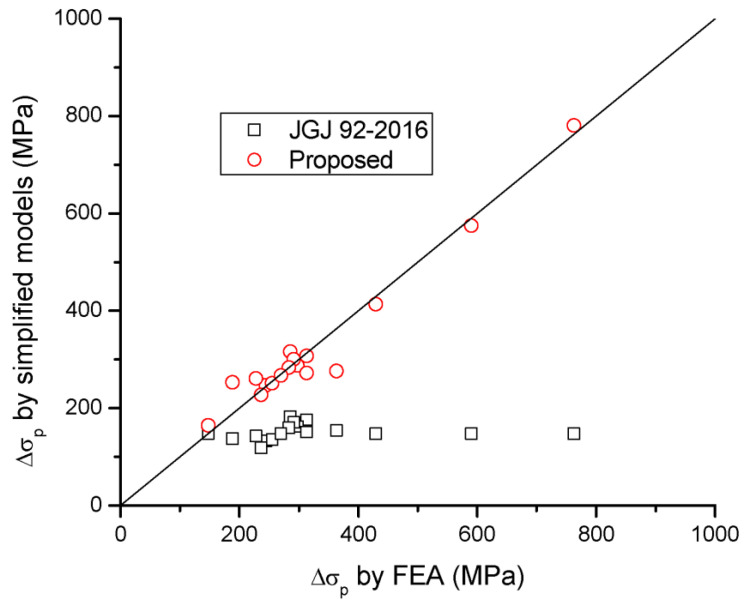
Correlation of Δ*σ_p_* by simplified models to FEA data.

**Figure 16:**
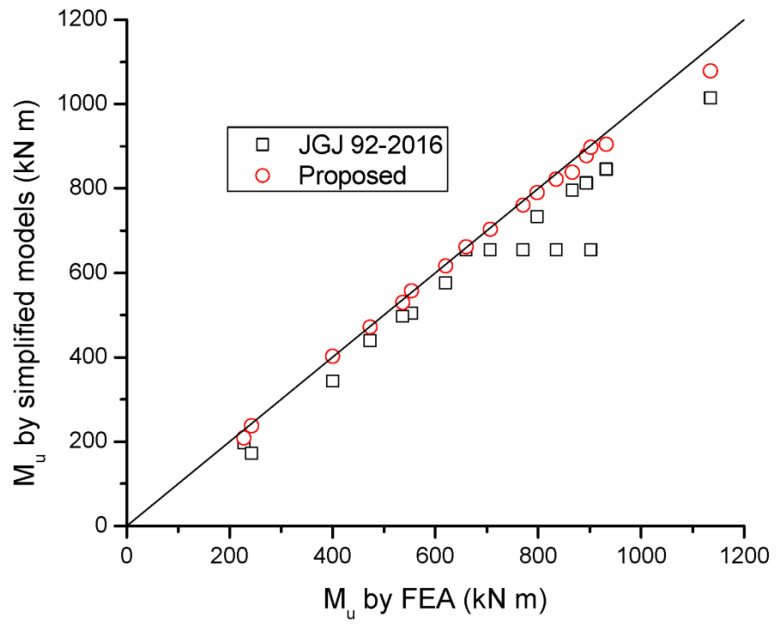
Correlation of *M_u_* by simplified models to FEA data.

A comparison of Δ*σ_p_* and *M_u_* for beams with different tendon-related variables predicted by the simplified models and FEA is presented in Table 5 and Figure 15 and Figure 16. It is seen that JGJ 92-2016 leads to significant underestimation in Δ*σ_p_*, and, therefore, this code underestimates the flexural strength of the beams. The predicted Δ*σ_p_* is 53.5% of the FEA data, on average, with a standard deviation of 16.2%, while the predicted *M_u_* is 88.6% of the FEA data, on average, with a standard deviation of 6.9%. The proposed simplified model shows much better predictions than JGJ 92-2016. According to the proposed model, the mean discrepancy for Δ*σ_p_* is 0.9%, with a standard deviation of 11.1%, while the mean discrepancy for *M_u_* is −1.6%, with a standard deviation of 2.1%.

## 5. Conclusions

By applying a validated FEA method, a numerical study is carried out to examine the flexural behavior of concrete beams prestressed with external CFRP tendons. Particular focus is placed on variables related to CFRP tendons, i.e., the area, prestress level, depth and elastic modulus of the tendons. An analytical model is also developed. The main conclusions are:

- CFRP tendons play a crucial role in the structural performance of externally prestressed beams, including the flexural stiffness, ultimate load-carrying capacity, stress increase in external tendons, deformation and ductility. A higher tendon area, initial prestress or elastic modulus causes a lower flexural ductility. The Δ*σ_p_* decreases as the tendon area or initial prestress level increases or as the tendon depth or elastic modulus increases.
- JGJ 92-2016 significantly underestimates the ultimate tendon stress, and hence, this code is over-conservative for flexural strength predictions of externally CFRP prestressed beams. The predicted Δ*σ_p_* and *M_u_* by JGJ 92-2016 are 53.5% and 88.6% of the FEA data, on average, respectively.
- An equation is proposed to calculate Δ*σ_p_*, considering the influence of the tendon area, effective prestress, tendon depth and modulus of elasticity. The proposed analytical model shows excellent predictions of tendon stress and flexural strength, i.e., the mean discrepancy for Δ*σ_p_* is 0.9% with a standard deviation of 11.1%, while the mean discrepancy for *M_u_* is −1.6% with a standard deviation of 2.1%.

## Figures and Tables

**Table 1:** Effect of tendon area on flexural ductility.

*A_p_* (mm^2^)	Deflection (mm)		Curvature (rad/mm)		Deflection Ductility	Curvature Ductility
	Yielding	Ultimate	Yielding	Ultimate		
200	21.9	123.4	5.3	43.3	5.63	8.16
650	43.2	119.1	5.9	34.3	2.75	5.78
1100	52.7	110.0	6.6	27.8	2.09	4.24
1550	57.3	102.7	7.0	20.0	1.79	2.86
2000	61.4	100.9	7.5	15.9	1.64	2.13

**Table 2:** Effect of initial prestress on flexural ductility.

*σ_p_*_0_ (MPa)	Deflection (mm)		Curvature (rad/mm)		Deflection Ductility	Curvature Ductility
	Yielding	Ultimate	Yielding	Ultimate		
0	18.6	116.5	5.0	40.8	6.25	8.13
368	25.8	118.7	5.6	36.3	4.61	6.53
736	45.8	115.2	6.1	32.0	2.51	5.25
1104	52.7	110.0	6.6	27.8	2.09	4.24
1472	56.5	105.0	6.9	22.0	1.86	3.18

**Table 3:** Effect of tendon depth on flexural ductility.

*d_p_* (mm)	Deflection (mm)		Curvature (rad/mm)		Deflection Ductility	Curvature Ductility
	Yielding	Ultimate	Yielding	Ultimate		
400	50.4	110.4	6.5	28.6	2.19	4.42
450	52.0	110.1	6.5	28.2	2.12	4.32
500	52.7	110.0	6.6	27.8	2.09	4.24
550	53.6	109.7	6.6	27.2	2.05	4.13
600	53.8	110.1	6.6	26.7	2.04	4.06

**Table 4:** Effect of tendon elastic modulus on flexural ductility.

*E_p_* (GPa)	Deflection (mm)		Curvature (rad/mm)		Deflection Ductility	Curvature Ductility
	Yielding	Ultimate	Yielding	Ultimate		
80	52.6	111.2	6.5	29.0	2.12	4.45
150	52.7	110.0	6.6	27.8	2.09	4.24
250	53.5	107.8	6.6	25.6	2.01	3.87
360	54.6	105.9	6.7	23.2	1.94	3.47
500	54.6	102.7	6.8	20.6	1.88	3.05

**Table 5:** Comparison of Δ*σ_p_* and *M_u_* by simplified models with FEA data.

*A_p_* (mm^2^)	*σ_p_*_0_ (MPa)	*d_p_* (mm)	*E_p_* (GPa)	Δ*σ_p_* (MPa)			*M_u_* (kN m)			(Δ*σ_p_*)*_sim_*/(Δ*σ_p_*)*_fea_*		(*M_u_*)*_sim_*/(*M_u_*)*_fea_*	
				JGJ	Pro	FEA	JGJ	Pro	FEA	JGJ	Pro	JGJ	Pro
200	1104	500	150	176	307	313	197	209	228	0.56	0.98	0.86	0.92
650				162	287	298	439	472	474	0.54	0.96	0.93	1.00
1100				147	267	270	655	703	707	0.55	0.99	0.93	0.99
1550				133	247	245	846	904	932	0.54	1.01	0.91	0.97
2000				118	227	237	1014	1078	1135	0.50	0.96	0.89	0.95
1100	0	500	150	182	316	285	172	238	243	0.64	1.11	0.71	0.98
	368			171	300	292	343	402	400	0.59	1.03	0.86	1.01
	736			159	283	283	504	558	554	0.56	1.00	0.91	1.01
	1104			147	267	270	655	703	707	0.55	0.99	0.93	0.99
	1472			135	251	255	795	838	867	0.53	0.98	0.92	0.97
1100	1104	400	150	137	253	188	497	530	537	0.73	1.35	0.93	0.99
		450		143	261	228	576	616	620	0.63	1.14	0.93	0.99
		500		147	267	270	655	703	707	0.55	0.99	0.93	0.99
		550		151	272	313	734	790	798	0.48	0.87	0.92	0.99
		600		154	276	363	813	877	894	0.42	0.76	0.91	0.98
1100	1104	500	80	147	164	148	655	661	660	1.00	1.11	0.99	1.00
			150	147	267	270	655	703	707	0.55	0.99	0.93	0.99
			250	147	414	429	655	761	770	0.34	0.96	0.85	0.99
			360	147	575	590	655	822	835	0.25	0.97	0.78	0.98
			500	147	780	763	655	897	902	0.19	1.02	0.73	0.99

Note: JGJ = JGJ 92-2016; Pro = proposed simplified model; (Δ*σ_p_*)*_sim_* and (Δ*σ_p_*)*_fea_* = ultimate stress increase in external tendons predicted by simplified models and by FEA, respectively; (*M_u_*)*_sim_* and (*M_u_*)*_fea_* = flexural strength by simplified models and FEA, respectively.

## Data Availability

Not applicable.
